# SCENet: Secondary Domain Intercorrelation Enhanced Network for Alleviating Compressed Poisson Noises

**DOI:** 10.3390/s19081939

**Published:** 2019-04-25

**Authors:** Seok Bong Yoo, Mikyong Han

**Affiliations:** IoT Research Division, Hyper-connected Communication Research Lab., Electronics Telecommunications Research Institute (ETRI), Daejeon 34129, Korea; mkhan@etri.re.kr

**Keywords:** compressed Poisson noises, signal-dependent noise property, intercorrelation, deep neural network

## Abstract

In real image coding systems, block-based coding is often applied on images contaminated by camera sensor noises such as Poisson noises, which cause complicated types of noises called compressed Poisson noises. Although many restoration methods have recently been proposed for compressed images, they do not provide satisfactory performance on the challenging compressed Poisson noises. This is mainly due to (i) inaccurate modeling regarding the image degradation, (ii) the signal-dependent noise property, and (iii) the lack of analysis on intercorrelation distortion. In this paper, we focused on the challenging issues in practical image coding systems and propose a compressed Poisson noise reduction scheme based on a secondary domain intercorrelation enhanced network. Specifically, we introduced a compressed Poisson noise corruption model and combined the secondary domain intercorrelation prior with a deep neural network especially designed for signal-dependent compression noise reduction. Experimental results showed that the proposed network is superior to the existing state-of-the-art restoration alternatives on classical images, the LIVE1 dataset, and the SIDD dataset.

## 1. Introduction with Preliminary Examination

**Figure 1:**
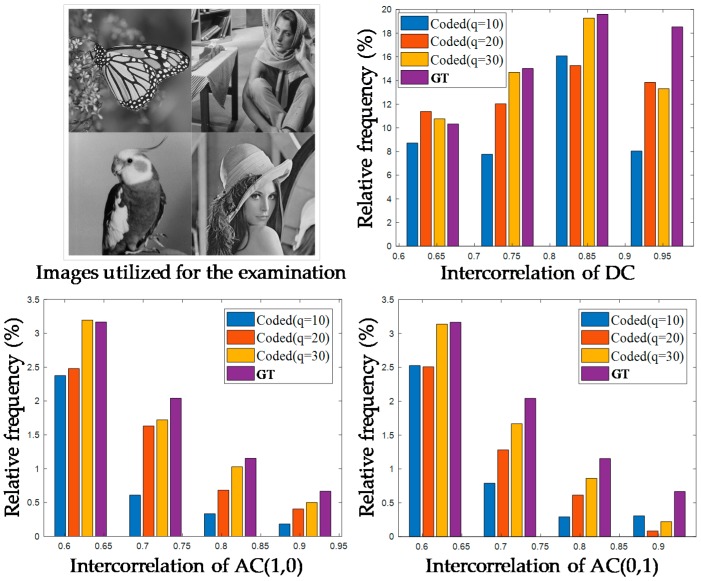
Intercorrelation distribution according to the compression level.

The block-based discrete cosine transform (BDCT) coding scheme is typically adopted for various coding standards such as JPEG, MPEG4, H.264/AVC, and H.265/HEVC for image and video compression. However, block-based coding suffers from well-known undesirable blocking artifacts due to the distortion of spatial correlation between neighboring blocks called intercorrelation. Meanwhile, according to [1], noises in real images captured by charge-coupled device (CCD) imaging sensors generally tend to have signal-dependent characteristics such as a Poisson distribution. Accordingly, the coding of the Poisson noise-corrupted image generates complex signal-dependent compressed noises that are called compressed Poisson noises. By applying recent compressed image restoration algorithms [2,3,4,5,6,7,8,9,10,11,12,13,14] based on convolutional neural networks (CNN) on the degraded image, it may be possible to reduce the conventional blocking and ringing artifacts. However, despite their promising solutions on conventional artifacts, the existing algorithms still do not provide excellent performance on compressed Poisson noises. This is because both an accurate image degradation model considering practical imaging systems and signal-dependent noise characteristics have not been seriously dealt with in the existing neural networks. To cope with the issues due to compressed Poisson noises, in this paper, we introduced an image degradation model suitable to the practical application and present a robust multi-band neural network to the signal-dependent noise property by exploiting a variance-stabilizing transformation (VST) [15]. In addition, the existing restoration algorithms [2,3,4,5,6,7,8,9,10,11,12,13,14] may not assure their best performance on coded images especially at low bit rates because the intercorrelation distortion recovery of each BDCT coefficient is not analytically reflected in the existing networks. In this paper, to verify the effect of the block-based coding on the intercorrelation, we performed a preliminary study by investigating the intercorrelation of each BDCT coefficient between neighboring blocks. To this end, we first prepared four ground truth (GT) images given in the top left of Figure 1 and produced their JPEG-coded images with quality factors, *q*, of 10, 20, and 30 corresponding to low bit rates. For the four GT images and their twelve coded images, we obtain the secondary block, **b**, which is composed of 4 × 4 BDCT coefficients [16]. We then computed the coefficient’s spatial correlation in each secondary block, or the intercorrelation *ρ* as

(1) $$\rho =\min\left(\left|\frac{R_{0,1}}{R_{0,0}}\right|,\;\left|\frac{R_{1,0}}{R_{0,0}}\right|\right)$$

where

(2) $$R_{x,y}=\frac{\sum_{m=0}^{3-x}\sum_{n=0}^{3-y}b(m,n)b(m+x,n+y)}{\left(4-x)(4-y\right)}.$$

Note that we investigated the intercorrelation using both GT images and the corresponding coded images, while the previous study [16] only used GT images. We further observed the change of the intercorrelation distribution according to the compression level of the coded images, unlike in an earlier study [17].

Figure 1 shows the distributions of the computed high intercorrelation values (*ρ* > 0.6) for the three lowest frequency (LF) BDCT coefficients: DC, AC(1,0), and AC(0,1). As the compression level increases, the relative frequencies of the high intercorrelation values for three LF coefficients commonly decreases. For example, the relative frequency of 63.4% for the DC of GT images decreases to 58.0%, 52.5%, and 40.5% in JPEG-coded images with *q* of 30, 20, and 10, respectively. The same tendency could also be observed for the other two LF coefficients, AC(1,0) and AC(0,1), while the remaining high-frequency (HF) coefficients did not necessarily follow this trend. From this examination, we considered that the intercorrelation enhancement of three LF coefficients was required for the effective restoration of coded images. In addition, the intercorrelation distortion of the BDCT coefficients occurred differently for each coefficient, as a different quantization step size was applied for each one in JPEG. Hence, in this paper, we propose an intercorrelation enhancement network in the secondary domain, which enabled us to improve the distorted intercorrelation of each frequency coefficient adaptively.

## 2. Degradation Model

**Figure 2:**
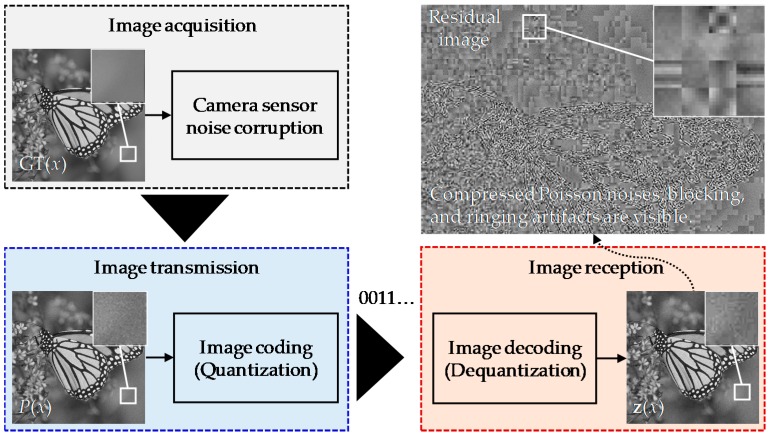
Image degradation model due to the coding of Poisson noisy images.

Considering the practical imaging systems described above, we defined a simple but effective image degradation model consisting of three procedures: camera sensor noise corruption, image coding based on quantization in the BDCT domain, and image decoding based on dequantization in the BDCT domain, as illustrated in Figure 2.

Here, let **z** be a decoded block in the receiver, specifically,

(3) $$z(x)=T^{-1}\left\{round\left\{\frac{T\left\{aP(x)\right\}}{Q}\right\}\times Q\right\}$$

where *x* denotes a spatial coordinate; and *T* and *T*^−1^ are the BDCT and inverse BDCT (IBDCT) operators, respectively. In addition, *P* denotes a Poisson variable scaled by *a* with a mean value *µ*, and **Q** denotes a quantization table. The probability distribution of the acquired value *P*(*x*) is derived as

(4) $$p(P(x)|\mu )=\frac{\mu^{P(x)}e^{-\mu }}{P(x)!}.$$

To better observe the visual image degradation of the received image **z**, a residual image can be obtained by subtracting the GT image from **z**, as shown in the top right of Figure 2. We note here that the decoded image suffers from complicated degradations including compressed Poisson noises as well as well-known blocking and ringing artifacts. It can also be observed that original near-random Poisson noises in *P*(*x*) were deformed to annoying patterns that had a strong spatial correlation, even in smooth regions.

## 3. Secondary Domain Intercorrelation Enhanced Network

**Figure 3:**
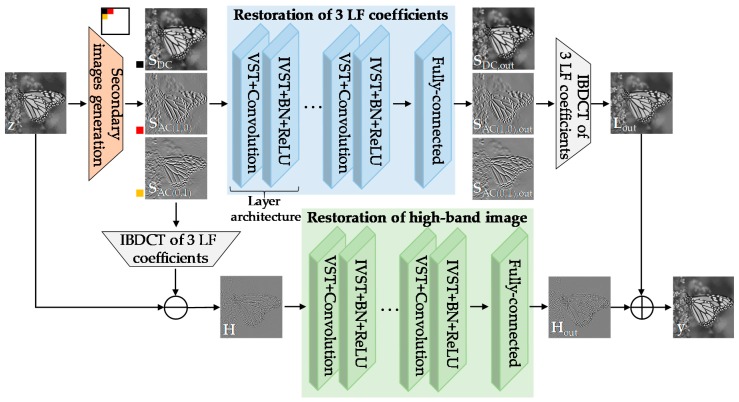
The architecture of the secondary domain intercorrelation enhanced network.

To reflect the defined image degradation model in the neural network adequately, in this paper, we suggested a secondary domain intercorrelation enhanced network (SCENet), which is quite suitable to address compressed Poisson noises, as illustrated in Figure 3. Inspired by our preliminary examination, we adapted and extended the secondary domain approach [17], which is still valuable for recovering the intercorrelation distortion. Note that the proposed algorithm has a clear difference from the existing algorithm [17] in terms of method and application. Specifically, while the classical edge-preserving total variation (TV) filtering was applied in the secondary domain for removing well-known blocking artifacts in [17], we utilized the deep neural network specially trained for reducing compressed Poisson noises, instead of the classical filtering. We also exploited the variance-stabilizing model to deal with signal-dependent noise characteristics, unlike in [17]. In other words, we combined key elements of the VST-based secondary domain intercorrelation model with a deep neural network that was particularly trained using the defined compressed Poisson noise model. The proposed SCENet architecture had two major parallel phases: restoration of the three LF coefficients and restoration of the high-band (HB) image.

In one of the parallel phases, we increased the intercorrelation of DC, AC(1,0), and AC(0,1) in the secondary domain, as shown at the top of Figure 3. In particular, the network had 20 layer architectures and each layer architecture was composed of five operations: VST, convolution, inverse VST (IVST), batch normalization (BN), and rectified linear units (ReLU). For an input image **z**, we first generated three secondary images: **S**_DC_, **S**_AC(1,0)_, and **S**_AC(0,1)_. To this end, we computed three LF BDCT coefficients in each 8 × 8 block by shifting the block pixel-by-pixel with overlapping and then merged them into each secondary image, respectively. After that, to remove the signal dependency of compressed noises, the secondary image pixel value *s* was stabilized to have homoscedastic variance via the Anscombe transformation [15]

(5) $$f(s)=\left\{ \begin{array}{cc} 2\sqrt{s+3/8}\;, & s\geq -3/8\\ 0\;, & \mathrm{otherwise} \end{array}\right..$$

As a subsequent procedure, the convolution was undertaken with *K* pre-trained filters, *a_L,F_*, with a size of *W* × *H*. Next, destabilization based on the IVST was applied in order to retrieve the original heteroskedastic variance as

(6) $$E\left[f\left(s\right)|\mu \right]=\sum_{i=0}^{\infty }\left(2\sqrt{s+3/8}\frac{\mu^{s}e^{-\mu }}{s!}\right).$$

**Figure 4:**
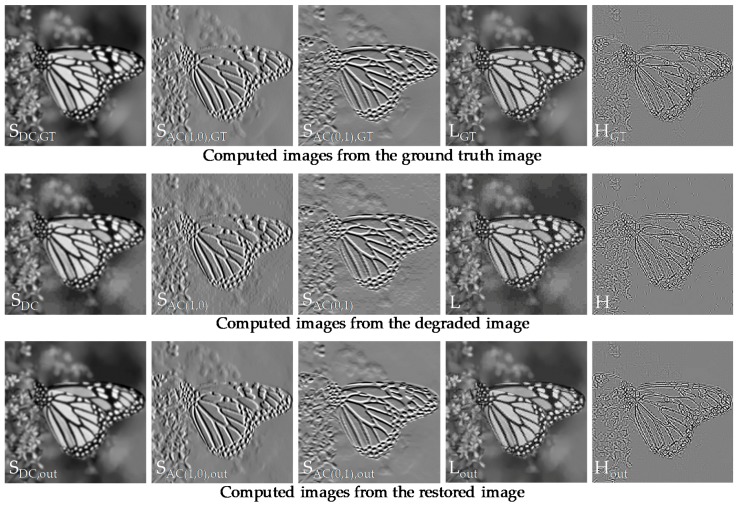
Stepwise restoration results of the SCENet. The first, second, and third columns show secondary images of DC, AC(1,0), and AC(0,1), respectively. The fourth and fifth columns show the LB and HB images, respectively.

The IVST step was then followed by BN and ReLU for fast and stable convergence in the training process. The iterative layer architectures were performed on three secondary images separately for the adaptive restoration of each coefficient that had different quantization amounts. Final feature maps were reshaped to the original input tensor size via a fully-connected layer and then an output low-band (LB) image **L**_out_ was obtained by applying *T*^−1^ to three filtered coefficients, **S**_DC,out_, **S**_AC(1,0),out_, and **S**_AC(0,1),out_, in each block without overlapping. The images from the first column to the fourth column of Figure 4 show that the three LF coefficients restoration network successfully recovered the secondary images and the LB image, similar to their corresponding GT images by addressing artifacts in degraded images.

In another parallel phase for restoring the HB image, we first obtained the input LB image **L** by applying *T*^−1^ to three LF coefficients in each 8 × 8 block. The input HB image **H** was then acquired by subtracting **L** from the input image **z**. Note that **H** corresponded to the remaining 61 HF coefficients in each block. Next, the filtered output **H**_out_ was computed via the same iterative layer architectures as in the restoration of the three LF coefficients by using convolution filters *b_L_* instead of *a_L,F_*. Note in the fifth column of Figure 4 that the network effectively recovered **H**_out_ quite close to the GT. The final restoration result could be obtained by adding two output images, **L**_out_ and **H**_out_. All of the above steps are also described in Algorithm 1.

**Algorithm 1:** Compressed Poisson noise reduction based on the SCENet**Input**: degraded image **z** and trained parameters *a*_*L*=1,...,20, *F*=1,2,3_, *b*_*L*=1,...,20_**Output**: restored image **y**1:  Compute DC, AC(1,0), AC(0,1) by *T*{**z**}.2:  Obtain **S** = {**S**_DC_, **S**_AC(1,0)_, **S**_AC(0,1)_} by merging the each coefficient.3:  **for**
*L* = 1, …, 20 **do**4:  Stabilize using VST by *f*{**S**}.5:  Apply convolution with trained parameters and then destabilize it by *f*
^−1^{a_L,F_* f{**S**}}.6:  Appy BN and ReLU by max(BN{*f*
^−1^{a_L,F_* f{**S**}}}, 0).7:  end for8:  Obtain **S**_out_ = {**S**_DC,out_, **S**_AC(1,0),out_, **S**_AC(0,1),out_} by applying a fully-connected layer.9:  Estimate **L**_out_ by *T*^−1^{**S**_out_}.10: Obtain **H** by **z** −*T*^−1^{**S**}.11: Estimate **H**_out_ by running steps 3−8 above with **H** and *b*_*L*=1,...,20_ instead of **S** and *a_L,F_*.12: Estimate final restored image by **y** = **L**_out_ + **H**_out_.

## 4. Experiments

**Figure 5:**
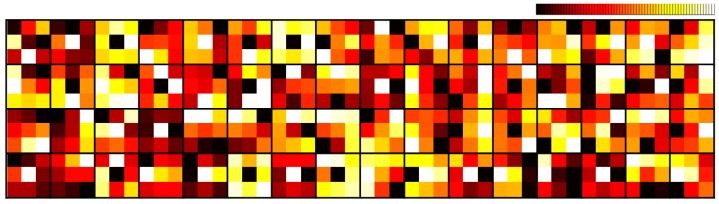
An example of convolution filter parameters that were obtained via our network training.

To train our networks, we used 400 images from the BSDS500 dataset [18] and 800 images from the DIV2K dataset [19]. Given a GT image, we synthesized three JPEG degraded images with different noise levels of {quality factor *q*, peak} = {10, 200}, {20, 400}, and {30, 600}. To generate Poisson noises, the maximum intensity of the GT image was first normalized to have the defined peak value. Next, the noise corruption was performed on the normalized image and the corrupted image was then denormalized to have the original maximum intensity. Therefore, the lower the peak value, the higher the Poisson noise level. In the restoration network of DC, AC(1,0), and AC(0,1), the sizes of *W* × *H* for filter parameters *a*_*L,F* = 1_, *a*_*L,F* = 2_, and *a*_*L,F* = 3_ were set to 3 × 3, 3 × 1, and 1 × 3, respectively, by considering the dominant pattern of compression artifacts in each secondary image. For example, the secondary image of AC(1,0) (or AC(0,1)) included only the vertical (or horizontal) artifacts affected by the BDCT basis function, as shown in the second row of Figure 4. In addition, the number of filters *K* in all networks was set to 64 and the *W* × *H* for the HB image restoration network parameters *b_L_* was empirically set to 3 × 3 in every architecture layer. Given a set of secondary images and HB images computed from GT images and their corresponding degraded images, we used the mean squared error (MSE) as a loss function. To minimize the loss function, we adopted an optimization method, Adam [20] with a batch size of 32. The learning rate was set to drop exponentially from 1e^−3^ to 1e^−5^. The proposed network was separately trained according to each noise level on one NVIDIA GTX 1080 GPU, under MATLAB R2017b with the MatConvNet package for about 16 h. Figure 5 shows an example of the convolution filters that were obtained via the network training. The whole inference time was about 120 ms for a 512 × 512 image and the time could be further reduced via parallel processing.

**Figure 6:**
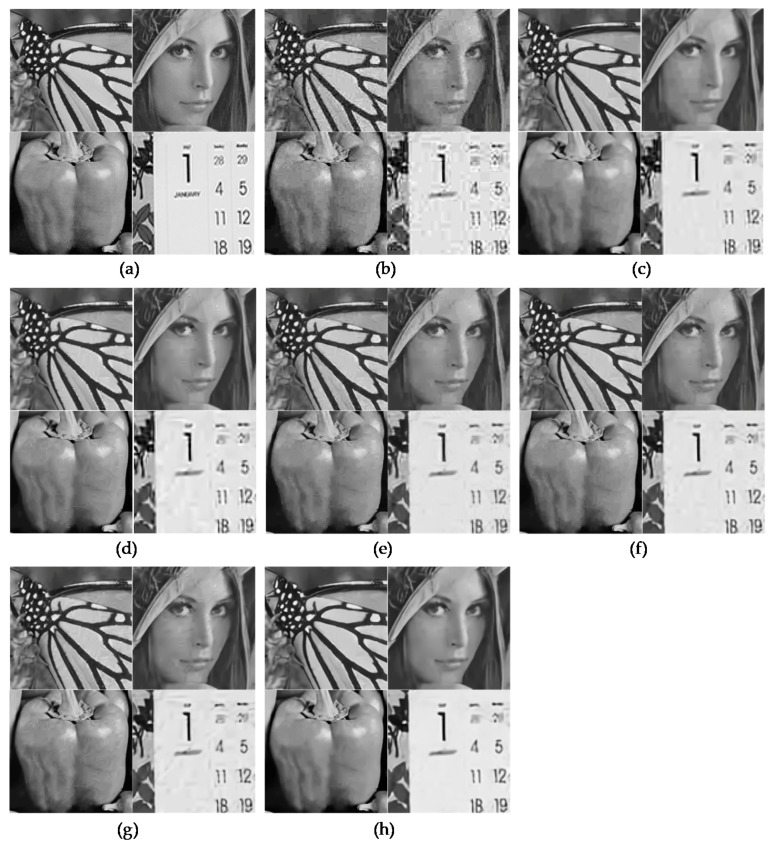
Comparison of the subjective visual quality for four classical images: Butterfly, Lena, Peppers, and Mobile. (**a**) Ground truths. (**b**) Degraded images with *q* = 10 and peak = 200. (**c**). Results of BM3D [22]. (**d**) Results of SSRQC [23]. (**e**) Results of ARCNN [2]. (**f**) Results of DnCNN [4]. (**g**) Results of MWCNN [5]. (**h**) Results of SCENet.

**Figure 7:**
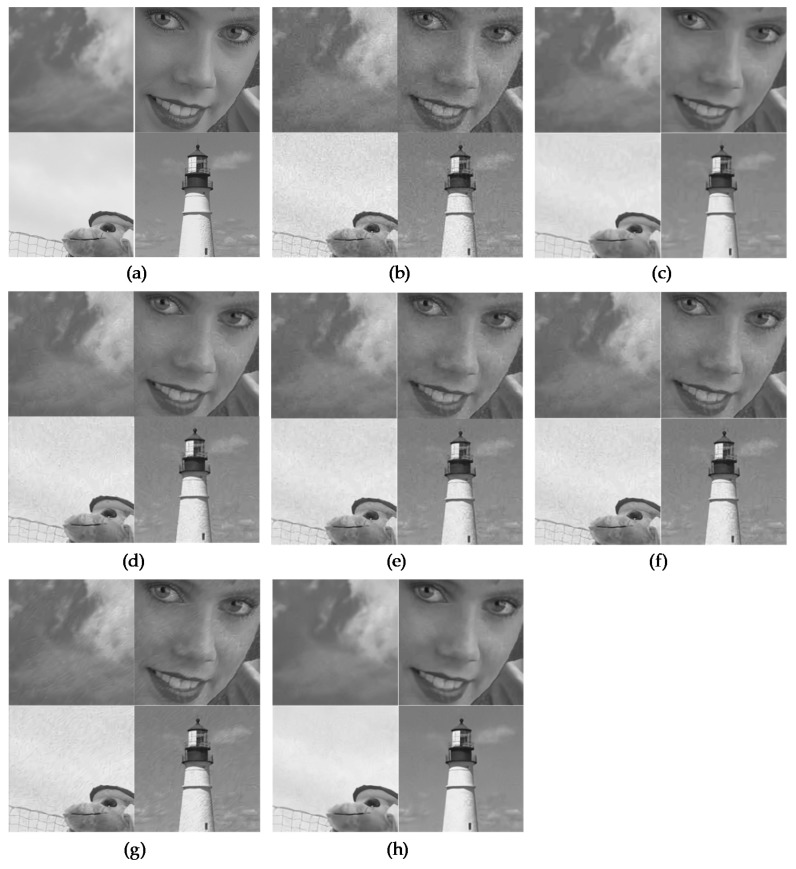
Comparison of the subjective visual quality for four images from the LIVE1 database: Caps, Womanhat, Carnivaldolls, and Lighthouse2. (**a**) Ground truths. (**b**) Degraded images with *q* = 20 and peak = 400. (**c**) Results of BM3D [22]. (**d**) Results of SSRQC [23]. (**e**) Results of ARCNN [2]. (**f**) Results of DnCNN [4]. (**g**) Results of MWCNN [5]. (**h**) Results of SCENet.

Meanwhile, in order to validate our trained networks, we used eight classical images given in Table 1 and 29 images from the LIVE1 dataset [21]. Figure 6 and Figure 7 show several restoration results for the JPEG degraded images with different noise levels of *q* and peak values to evaluate the subjective performance of the proposed network. We also compared the performance with a general compression artifact reduction algorithm [2] and two existing state-of-the-art restoration algorithms [4,5] based on CNN. The two existing denoising algorithms [22,23] that were not based on CNN were additionally used for the comparison. Open source codes in the first authors’ websites were applied for the comparison. The pre-trained models for MWCNN [5] were kindly provided by P. Liu, because it was not accessible via that website. We can easily note that in Figure 6 and Figure 7 that the results of the existing algorithms were not satisfactory because the undesirable compressed Poisson noises still remained, especially in many flat regions such as the wing, the face, the pepper, the calendar, the sky, and the wall. In contrast, the proposed SCENet provided more visually pleasing images by successfully alleviating the annoying compressed Poisson noises while preserving the image details in comparison to the existing algorithms. This noticeable visual improvement was achieved by the proposed VST-based secondary domain intercorrelation prior that was enforced in the neural network. The full resolution image results and an executable program for reproducing the results are also available on our website [24].

In addition to the subjective comparison, a quantitative comparison was conducted. Table 1 summarizes the peak signal-to-noise ratio (PSNR) and structure similarity (SSIM) [25] values computed from the processed results of the eight classical test images. It can be noticed in the table that the proposed SCENet provided the best objective quality except for only one case, by restoring the GT pixel values well. The objective comparison on the LIVE1 dataset was additionally conducted, as given in Table 2. The average PSNR and SSIM values were calculated from the luminance channels of 29 images in the dataset. This demonstrates that the proposed network overall outperformed the existing compressed image restoration algorithms as well as providing significant quality improvement when compared with the input degraded images.

**Figure 8:**
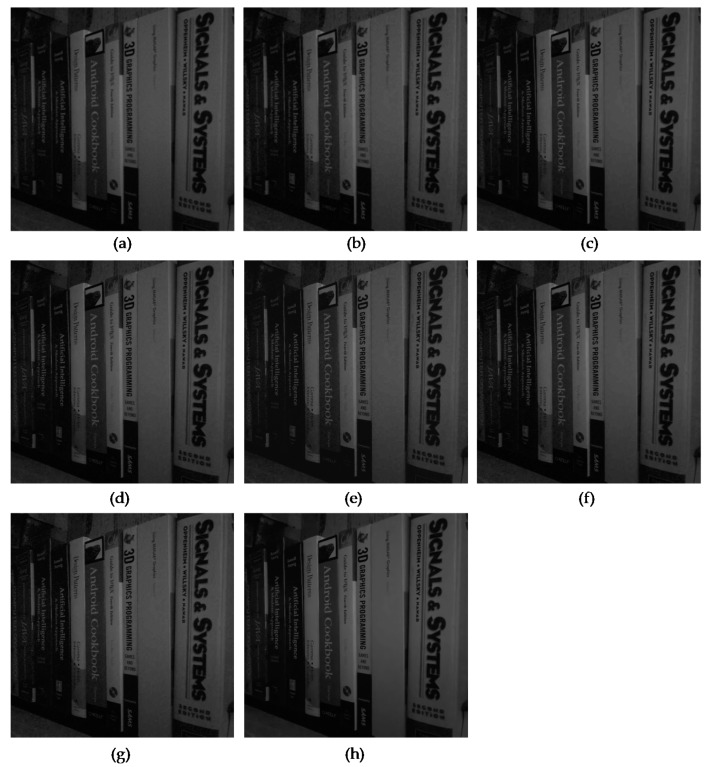
Comparison of the subjective visual quality for the image from the SIDD dataset, Books. (**a**) Captured image using an iPhone 7 with ISO = 800 and exposure = 1/2000 (BRISQUE = 43.36). (**b**) JPEG-coded image with *q* = 30 (BRISQUE = 41.97). (**c**) Result of BM3D [22] (BRISQUE = 45.06). (**d**) Result of SSRQC [23] (BRISQUE = 37.01). (**e**) Result of ARCNN [2] (BRISQUE = 33.15). (**f**) Result of DnCNN [4] (BRISQUE = 33.09). (**g**) Result of MWCNN [5] (BRISQUE = 37.65). (**h**) Result of SCENet (BRISQUE = 25.55).

**Figure 9:**
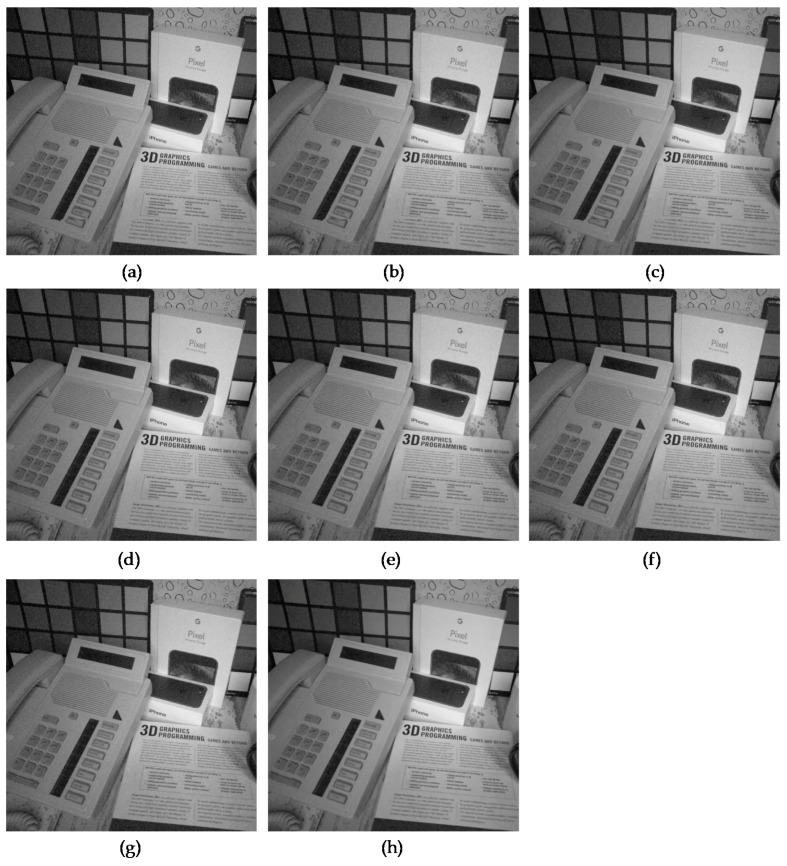
Comparison of the subjective visual quality for the image from the SIDD dataset, Desk. (**a**) Captured image using an iPhone 7 with ISO = 200 and exposure = 1/400 (BRISQUE = 46.81). (**b**) JPEG-coded image with *q* = 30 (BRISQUE = 28.98). (**c**) Result of BM3D [22] (BRISQUE = 22.20). (**d**) Result of SSRQC [23] (BRISQUE = 26.07). (**e**) Result of ARCNN [2] (BRISQUE = 24.35). (**f**) Result of DnCNN [4] (BRISQUE = 21.78). (**g**) Result of MWCNN [5] (BRISQUE = 24.11). (**h**) Result of SCENet (BRISQUE = 19.70).

In addition, to evaluate the restoration performance of several algorithms on the actual sensor noises, we used the smartphone image denoising dataset (SIDD) [26] because smartphone images tend to have notably severe Poisson noises due to the small aperture and sensor size. Figure 8 and Figure 9 show the comparison of the algorithms for two images from the SIDD dataset, Books and Desk, respectively. The two images were acquired using an iPhone 7 with different camera settings, ISO, and exposure time and they included real camera sensor noises, as shown in Figure 8a and Figure 9a. The JPEG compression of the sensor noises generates compressed Poisson noises, as shown in Figure 8b and Figure 9b. We note that the proposed SCENet alleviated the compressed Poisson noises well, as shown in Figure 8h and Figure 9h, while the results of the existing algorithms still included the noises in the book, the phone, the paper, and the box, as shown in Figure 8c–g and Figure 9c–g. As their original GT images were not available, we conducted an objective comparison using a no-reference image quality metric, the blind/referenceless image spatial quality evaluator (BRISQUE) index [27]. The lower the BRISQUE values, the better the image quality. As expected in visual results in Figure 8 and Figure 9, we noticed that SCENet outperformed the existing algorithms on the real noise data in terms of the BRISQUE values.

## 5. Conclusions

Compressed Poisson noises are critical and troublesome issues generated in real image coding systems. To address sensor noises effectively, we analyzed the intercorrelation distortion process via our preliminary examination and proposed a new multi-band intercorrelation increment network that exploits the secondary domain instead of the typical spatial domain. Additionally, to increase robustness to the signal-dependent noise characteristics, we designed a layer architecture composed of five operations and trained the network parameters under the challenging image degradation model. The superior performance of the proposed network on three datasets was also validated in terms of both subjective and objective qualities.

## Figures and Tables

**Table 1:** Comparison of the objective quality on classical images in terms of PSNR (dB) and SSIM. Bold values indicate the best scores.

Method	Noise Level	Metric	Camera Man	Barbara	Bird	Butter Fly	Hall Monitor	Lena	Mobile	Peppers
Degraded	*q* = 10, peak = 200	PSNR	25.85	25.13	30.86	27.90	27.91	29.89	21.85	29.39
		SSIM	0.737	0.753	0.805	0.836	0.771	0.777	0.750	0.754
	*q* = 20, peak = 400	PSNR	27.67	27.46	32.84	30.17	30.28	31.82	24.16	31.15
		SSIM	0.776	0.827	0.827	0.873	0.815	0.819	0.822	0.797
	*q* = 30, peak = 600	PSNR	28.97	29.17	34.02	31.41	31.56	32.84	25.73	32.04
		SSIM	0.814	0.864	0.850	0.888	0.839	0.841	0.859	0.819
BM3D [22]	*q* = 10, peak = 200	PSNR	27.22	27.18	33.13	29.97	29.89	31.29	22.83	31.53
		SSIM	0.809	0.805	0.901	0.913	0.858	0.802	0.782	0.825
	*q* = 20, peak = 400	PSNR	29.16	29.69	35.70	32.38	32.20	33.08	25.43	32.93
		SSIM	0.846	0.880	0.920	0.932	0.899	0.840	0.876	0.842
	*q* = 30, peak = 600	PSNR	30.47	31.21	37.14	33.59	33.75	34.29	26.95	33.27
		SSIM	0.871	0.902	0.921	0.931	0.902	0.858	0.897	0.844
SSRQC [23]	*q* = 10, peak = 200	PSNR	27.32	27.31	33.14	30.53	29.73	31.27	23.05	31.49
		SSIM	0.814	0.821	0.891	0.913	0.853	0.805	0.794	0.818
	*q* = 20, peak = 400	PSNR	28.97	29.32	35.28	32.65	32.20	32.94	25.29	32.91
		SSIM	0.844	0.872	0.905	0.930	0.880	0.835	0.864	0.836
	*q* = 30, peak = 600	PSNR	30.18	30.91	36.38	33.63	33.36	34.27	26.74	33.61
		SSIM	0.868	0.898	0.915	0.936	0.891	0.850	0.892	0.847
ARCNN [2]	*q* = 10, peak = 200	PSNR	27.34	26.63	33.33	31.04	30.01	31.88	23.22	31.27
		SSIM	0.819	0.811	0.901	0.918	0.880	0.847	0.807	0.827
	*q* = 20, peak = 400	PSNR	28.86	29.18	35.11	33.22	32.49	33.56	25.70	32.57
		SSIM	0.838	0.873	0.906	0.931	0.898	0.872	0.868	0.848
	*q* = 30, peak = 600	PSNR	30.15	30.85	36.26	34.37	33.76	34.43	27.22	33.38
		SSIM	0.860	0.902	0.912	0.937	0.907	0.885	0.897	0.860
DnCNN [4]	*q* = 10, peak = 200	PSNR	27.81	27.15	33.50	31.17	30.52	31.92	23.60	31.55
		SSIM	0.833	0.817	0.903	0.919	0.886	0.844	0.809	0.823
	*q* = 20, peak = 400	PSNR	29.58	29.63	35.33	33.52	32.94	33.63	26.22	32.95
		SSIM	0.852	0.873	0.909	0.934	0.901	0.867	0.875	0.839
	*q* = 30, peak = 600	PSNR	30.75	31.18	36.35	34.62	33.95	34.44	27.86	33.61
		SSIM	0.870	0.897	0.913	0.938	0.905	0.877	0.906	0.847
MWCNN [5]	*q* = 10, peak = 200	PSNR	28.09	**27.71**	33.23	31.10	30.62	31.91	23.69	31.53
		SSIM	0.830	0.820	0.894	0.918	0.880	0.845	0.809	0.821
	*q* = 20, peak = 400	PSNR	29.68	29.92	34.85	33.26	32.74	33.35	26.15	32.72
		SSIM	0.839	0.871	0.889	0.927	0.886	0.856	0.871	0.828
	*q* = 30, peak = 600	PSNR	30.98	31.55	36.07	34.54	34.00	34.32	27.74	33.49
		SSIM	0.864	0.898	0.900	0.931	0.897	0.870	0.900	0.841
SCENet	*q* = 10, peak = 200	PSNR	**28.24**	27.67	**34.08**	**31.72**	**31.13**	**32.37**	**23.90**	**32.12**
		SSIM	**0.846**	**0.825**	**0.916**	**0.932**	**0.905**	**0.851**	**0.822**	**0.835**
	*q* = 20, peak = 400	PSNR	**30.20**	**30.32**	**36.41**	**34.21**	**33.85**	**34.25**	**26.54**	**33.68**
		SSIM	**0.880**	**0.887**	**0.935**	**0.951**	**0.930**	**0.879**	**0.893**	**0.856**
	*q* = 30, peak = 600	PSNR	**31.36**	**31.84**	**37.58**	**35.37**	**35.10**	**35.18**	**28.17**	**34.43**
		SSIM	**0.894**	**0.910**	**0.939**	**0.955**	**0.936**	**0.891**	**0.920**	**0.866**

**Table 2:** Comparison of the objective quality on the LIVE1 database in terms of average PSNR (dB) and SSIM. Bold values indicate the best scores.

Noise Level	Metric	Degraded	BM3D [22]	SSRQC [23]	ARCNN [2]	DnCNN [4]	MWCNN [5]	SCENet
*q* = 10, peak = 200	PSNR	27.01	27.85	28.03	28.16	28.60	28.64	**28.97**
	SSIM	0.730	0.761	0.762	0.774	0.797	0.795	**0.805**
*q* = 20, peak = 400	PSNR	28.99	30.15	29.88	30.22	30.60	30.47	**31.08**
	SSIM	0.794	0.833	0.835	0.843	0.850	0.841	**0.863**
*q* = 30, peak = 600	PSNR	30.18	31.38	31.30	31.35	31.58	31.63	**32.34**
	SSIM	0.827	0.861	0.859	0.868	0.873	0.871	**0.890**

## References

[1] Foi A., Trimeche M., Katkovnik V., Egiazarian K. (2008). Practical Poissonian-Gaussian noise modeling and fitting for single-image raw-data. IEEE Trans. Image Process..

[2] Dong C., Deng Y., Change L.C., Tang X. Compression artifacts reduction by a deep convolutional network. Proceedings of the IEEE International Conference on Computer Vision.

[3] Chen Y., Pock T. (2017). Trainable nonlinear reaction diffusion: A flexible framework for fast and effective image restoration. IEEE Trans. Pattern Anal. Mach. Intell..

[4] Zhang K., Zuo W., Chen Y., Meng D., Zhang L. (2017). Beyond a Gaussian denoiser: Residual learning of deep CNN for image denoising. IEEE Trans. Image Process..

[5] Liu P., Zhang H., Zhang K., Lin L., Zuo W. Multi-level wavelet-CNN for image restoration. Proceedings of the IEEE Conference on Computer Vision and Pattern Recognition Workshops.

[6] Lu G., Ouyang W., Xu D., Zhang X., Gao Z., Sun M.T. Deep Kalman filtering network for video compression artifact reduction. Proceedings of the European Conference on Computer Vision.

[7] Zhang Y., Sun L., Yan C., Ji X., Dai Q. (2018). Adaptive residual networks for high-quality image restoration. IEEE Trans. Image Process..

[8] Zhang X., Yang W., Hu Y., Liu J. DMCNN: Dual-domain multi-scale convolutional neural network for compression artifacts removal. Proceedings of the 25th IEEE International Conference on Image Processing.

[9] Zheng B., Chen Y., Tian X., Zhou F., Liu X. (2018). Implicit dual-domain convolutional network for robust color image compression artifact reduction. arXiv.

[10] Zhang X., Lu Y., Liu J., Dong B. (2018). Dynamically unfolding recurrent restorer: A moving endpoint control method for image restoration. arXiv.

[11] Zheng B., Sun R., Tian X., Chen Y. (2018). S-Net: A scalable convolutional neural network for JPEG compression artifact reduction. J. Electron. Imaging.

[12] Yoo J., Lee S.H., Kwak N. Image restoration by estimating frequency distribution of local patches. Proceedings of the IEEE Conference on Computer Vision and Pattern Recognition Workshops.

[13] Galteri L., Seidenari L., Bertini M., Bimbo A.D. (2019). Deep universal generative adversarial compression artifact removal. IEEE Trans. Multimedia.

[14] Chen H., He X., An C., Nguyen T.Q. (2019). Deep wide-activated residual network based joint blocking and color bleeding artifacts reduction for 4: 2: 0 JPEG-compressed images. IEEE Signal Process. Lett..

[15] Markku M., Foi A. (2011). Optimal inversion of the Anscombe transformation in low-count Poisson image denoising. IEEE Trans. Image Process..

[16] Lim K.W., Chun K.W., Ra J.B. (1995). Improvements on image transform coding by reducing interblock correlation. IEEE Trans. Image Process..

[17] Yoo S.B., Choi K., Ra J.B. (2014). Post-processing for blocking artifact reduction based on inter-block correlation. IEEE Trans. Multimedia.

[18] Martin D., Fowlkes C., Tal D., Malik J. A database of human segmented natural images and its application to evaluating segmentation algorithms and measuring ecological statistics. Proceedings of the IEEE International Conference on Computer Vision.

[19] Agustsson E., Timofte R. Ntire 2017 challenge on single image super-resolution: Dataset and study. Proceedings of the IEEE Conference on Computer Vision and Pattern Recognition Workshops.

[20] Kingma D.P., Adam B.J. (2014). Adam: A method for stochastic optimization. arXiv.

[21] LIVE Image Quality Assessment Database Release 2. http://live.ece.utexas.edu/research/quality.

[22] Dabov K., Foi A., Katkovnik V., Egiazarian K. (2007). Image denoising by sparse 3-D transform-domain collaborative filtering. IEEE Trans. Image Process..

[23] Zhao C., Zhang J., Ma S., Fan X., Zhang Y., Gao W. (2017). Reducing image compression artifacts by structural sparse representation and quantization constraint prior. IEEE Trans. Circuits Sys. Video Technol..

[24] SCENet Program. https://github.com/seokbongyoo/SCENet.

[25] Wang Z., Bovik A.C., Sheikh H.R., Simoncelli E.P. (2004). Image quality assessment: From error visibility to structural similarity. IEEE Trans. Image Process..

[26] Smartphone Image Denoising Dataset. https://www.eecs.yorku.ca/~kamel/sidd/index.php.

[27] Mittal A., Moorthy A.K., Bovik A.C. (2012). No-reference image quality assessment in the spatial domain. IEEE Trans. Image Process..

